# Safety and effectiveness of secukinumab subcutaneous injection in Japanese patients with psoriasis vulgaris and psoriatic arthritis: A post‐marketing surveillance

**DOI:** 10.1111/1346-8138.17499

**Published:** 2024-11-29

**Authors:** Ayako Fujishige, Noriko Seko

**Affiliations:** ^1^ Department of Japan CD Management and Rex‐Management Novartis Pharma K.K Tokyo Japan; ^2^ Biostatistics Novartis Pharma K.K Tokyo Japan

**Keywords:** effectiveness, psoriasis vulgaris, psoriatic arthritis, safety, secukinumab

## Abstract

Secukinumab is the first human monoclonal antibody that targets human interleukin‐17A. This open‐label, multicenter, uncontrolled, single‐arm, prospective observational surveillance evaluated the long‐term safety and effectiveness of secukinumab in patients with psoriasis vulgaris and psoriatic arthritis (PsA) in Japan. Of 997 patients whose surveillance forms were collected, 976 were included in the safety analysis and 729 in the effectiveness analysis. Prior to the start of secukinumab treatment, biologics were used in 42.52% of patients for the treatment of conditions including psoriasis. The mean ± standard deviation (SD) duration of secukinumab administration was 288.1 ± 106.51 days and the median (range) was 344.0 (1–365) days. The most commonly used dose per administration was 300 mg in 96.21% (939 patients) and the mean ± SD total number of administrations was 13.6 ± 3.87. Adverse events (AEs), AEs suspected to be related to secukinumab, AEs that led to secukinumab treatment discontinuation, serious AEs, and deaths were reported in 36.17%, 18.85%, 8.09%, 5.84%, and 1.13%, respectively. The proportion of patients with an Investigator's Global Assessment score improvement to 0/1 increased over time from the start of secukinumab treatment to week 24 and remained stable thereafter. The Psoriasis Area and Severity Index 75 response rates and the proportions of patients with a Dermatology Life Quality Index score of 0/1 increased from baseline and were maintained up to week 52. This surveillance did not show any new safety concerns of secukinumab treatment. The effectiveness of secukinumab treatment was observed in patients with psoriasis vulgaris and PsA.

## 1 INTRODUCTION

Psoriasis is a common skin disease with a prevalence of approximately 0.3% in Japan.^1^ The common types of psoriasis include psoriasis vulgaris, with a classic presentation of sharply demarcated pinkish/reddish plaques with overlying white/silver scales, and psoriatic arthritis (PsA), a chronic, heterogeneous disorder characterized by inflammatory arthritis in psoriatic patients.^2^ In a survey conducted by the Japanese Society for Psoriasis Research, the prevalence of PsA among psoriatic patients was estimated to be 15.3%.^3^

Biologics targeting different cytokines have shown improvement in the treatment of psoriasis and, as per the Japanese guidance for use of biologics for psoriasis (2022), 11 biologics have been approved in Japan for the indication of psoriasis.^4^ In psoriasis, interleukin (IL)‐17A is involved in the formation and maintenance of the pathological conditions through potentiation with tumor necrosis factor alpha (TNFα), interferon γ, and other cytokines. IL‐17A, produced in T helper (Th)‐17 cells, mast cells, and neutrophils, and directly activates keratinocytes and skin fibroblasts to facilitate the production of cytokines, chemokines, and antimicrobial peptides.^5^ Secukinumab is the first human immunoglobulin (Ig)G1/κ monoclonal antibody in the world targeting human IL‐17A. It is approved for the indication of psoriasis vulgaris or PsA with inadequate response to conventional therapies in Japan since December 2014.^6^, ^7^

Limited Japanese data are available from the clinical studies conducted to register the psoriasis or PsA indication of secukinumab.^8^, ^9^, ^10^ A previous study by Fujita et al. (2021) presented the retrospective data since the launch of secukinumab but did not include prospective study data.^6^ The present study is a post‐marketing surveillance conducted as instructed by the Japanese regulatory authorities to collect post‐marketing data of secukinumab. This surveillance aimed to evaluate the safety and effectiveness of secukinumab in clinical use in patients with psoriasis vulgaris or PsA.

## 2 METHODS

### 2.1 Study design

This was an open‐label, multicenter, single‐arm, prospective observational surveillance to evaluate the safety and effectiveness of secukinumab in patients with psoriasis vulgaris or PsA. Patients received secukinumab in accordance with the Good Post‐marketing Study Practice. The investigator filled out and signed the surveillance forms for all eligible patients (including patients who discontinued). Each patient was observed for 52 weeks from the first secukinumab dose and followed up for 2 years (156 weeks from the start of secukinumab treatment) after the end of the observation period for the exclusive collection of data on the specific adverse events (AEs) that were determined by the regulatory authority.

### 2.2 Study population

All patients provided written informed consent prior to participation in this surveillance. Patients with either psoriasis vulgaris or PsA who were inadequately responding to conventional therapies and receiving secukinumab for the first time or patients with refractory eruption or joint symptoms were included in the surveillance from November 4, 2015. Patients previously treated or planned to be treated with a product containing the same active ingredient as secukinumab (either as an investigational drug or in a post‐marketing clinical study) were excluded.

### 2.3 Safety analysis

The safety analysis population consisted of patients who were eligible for this surveillance but excluded those who failed to return after the first dose of secukinumab, who did not receive secukinumab, whose first surveillance forms were not locked, who were unregistered, whose surveillance forms were invalid, and those with unknown AE status. Safety assessments included the incidence of AEs, AEs that led to secukinumab treatment discontinuation, serious AEs (SAE), AEs suspected by investigators to be related to secukinumab, and events that corresponded to the priority investigation items. The priority investigation items were defined as the identified risks or potential risks in the secukinumab risk management plan (i.e., serious infections, tuberculosis, neutropenia, fungal infections, hypersensitivity reactions, malignant tumors, inflammatory bowel disease [IBD], and cardiovascular/cerebrovascular events). These events, along with the severity and the relatedness of AEs to secukinumab, were assessed at the investigators' discretion. Odds ratios (ORs) of the occurrence of AEs suspected to be related to secukinumab between the categories of characteristics and their two‐sided 95% confidence intervals (CIs) were calculated. Univariate and multivariate logistic regression analyses were performed for factors where the 95% confidence interval of the odds ratio did not include 1, as well as for factors suspected to have a clinical relationship.

### 2.4 Effectiveness analysis

The effectiveness analysis population consisted of patients in the safety analysis population, excluding patients with no Investigator's Global Assessment (IGA) results or unevaluable results at all time points. Descriptive statistics were used to summarize effectiveness variables and provided for patients who were evaluable at each time point. The proportions of patients with an IGA score improvement to 0 (clear) or 1 (almost clear), Psoriasis Area and Severity Index (PASI) 75/90/100 response rates, and a Dermatology Life Quality Index (DLQI) total score of 0 or 1 at the respective evaluation time points were calculated together with the 95% CIs.

The following variables were evaluated only for patients diagnosed with PsA at the start of secukinumab treatment: Disease Activity Score‐28‐C‐reactive protein (DAS‐28‐CRP), Health Assessment Questionnaire©—Disability Index (HAQ‐DI), Global Impression of Change in joint symptoms, tender and swollen distal interphalangeal (DIP) joint counts in fingers and toes, tenderness in entheses, pain on visual analog scale (VAS) by patient, pain on VAS by physician, dactylitis counts in fingers and toes, and Bath Ankylosing Spondylitis Disease Activity Index (BASDAI) score.

For the patients with baseline IGA score of ≥2 in the effectiveness analysis population, analysis was performed to identify the characteristics that possibly affected the effectiveness (proportion of patients with an IGA score improvement to 0 or 1 at week 52). The ORs of the responders between the categories of characteristics and their two‐sided 95% CIs were calculated. Univariate and multivariate logistic regression analyses were performed for factors where the 95% confidence interval of the odds ratio did not include 1, as well as for factors suspected to have a clinical relationship.

## 3 RESULTS

### 3.1 Patient disposition and baseline characteristics

**FIGURE 1:**
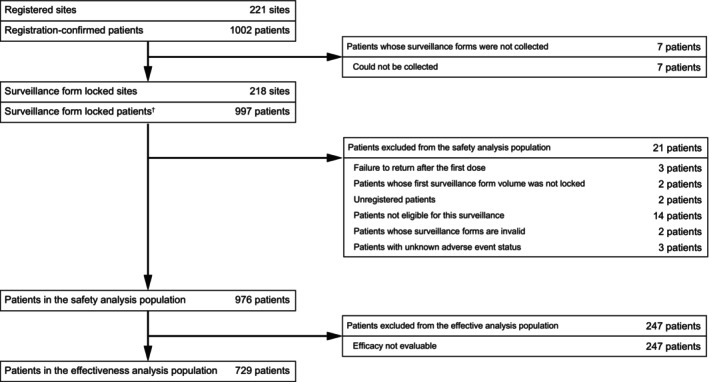
Patient disposition. ^†^Includes two patients whose surveillance forms were collected without registration confirmation. These two patients were regarded as unregistered patients and thus excluded from the safety analysis population. Patients with multiple reasons for exclusion were counted in each of the reasons for exclusion.

The data of 997 patients from 218 sites were collected (Figure 1). The patient baseline demographics and disease characteristics were analyzed for the safety analysis population (*n* = 976) and are presented in Table 1. The majority of the patients were aged ≥18 years (99.08%, *n* = 967/976) and the remaining patients were <18 years (0.92%, *n* = 9/976). Most of the patients had body mass index ≥18.5 kg/m^2^ to <25.0 kg/m^2^ (35.76%) and 25.0 kg/m^2^ to <35.0 kg/m^2^ (28.38%), followed by <18.5 kg/m^2^ (4.00%) and ≥35.0 kg/m^2^ (2.15%); others were unknown. Comorbidities were present in 56.35% of the patients (*n* = 550/976), and the common (>10%) medical conditions were cardiovascular/cerebrovascular events (18.85%) and hepatic impairment (13.01%), followed by renal disease (7.79%), hypersensitivity reactions (4.51%), and fungal infections (3.79%). Other comorbidities that were present in the patients were less than 1%.

**TABLE 1:** Patient demographics and baseline characteristics (safety analysis set).

Characteristic	Category/summary statistics	*N* = 976
Sex, *n* (%)	Male	681 (69.77)
	Female	295 (30.23)
Age (years)	Mean ± SD	54.1 ± 14.90
	Median (range)	53.0 (15–89)
Reasons for secukinumab use, *n* (%)	Psoriasis vulgaris	699 (71.62)
	PsA	277 (28.38)
Visit category, *n* (%)	Outpatient	942 (96.52)
	Inpatient	34 (3.48)
Total years of psoriasis vulgaris diagnosis, *n* (%)	<1 year	44 (4.51)
	≥1 to <5 years	135 (13.83)
	≥5 to <10 years	176 (18.03)
	≥10 to <20 years	328 (33.61)
	≥20 to <30 years	150 (15.37)
	≥30 years	98 (10.04)
	Unknown/not recorded	45 (4.61)
PsA duration (secukinumab use for PsA treatment), *n* (%)	<1 year	56 (5.74)
	≥1 to <5 years	94 (9.63)
	≥5 to <10 years	45 (4.61)
	≥10 to <20 years	44 (4.51)
	≥20 to <30 years	14 (1.43)
	≥30 years	3 (0.31)
	Unknown/not recorded	21 (2.15)
Weight (kg), *n* = 740	Mean ± SD	68.96 ± 16.110
	Median (range)	67.65 (32.0–163.0)
BMI (kg/m^2^), *n* = 686	Mean ± SD	25.02 ± 4.832
	Median (range)	24.39 (14.4–55.1)
Drinking habit, *n* (%)	Habitual drinker (drinking on a daily basis)	202 (20.70)
	Social drinker	243 (24.90)
	Non‐drinker	182 (18.65)
	Unknown/not recorded	349 (35.76)
Smoking history, *n* (%)	Current smoker	208 (21.31)
	Ex‐smoker (no longer a smoker)	159 (16.29)
	Non‐smoker	288 (29.51)
	Unknown/not recorded	321 (32.89)
Medical history, *n* (%)	Yes	342 (35.04)
Current medical conditions, *n* (%)	Yes	550 (56.35)
Previous drugs, *n* (%)	Yes	888 (90.98)
Biological products (for psoriasis treatment), *n* (%)	Yes	413 (42.52)
Drugs for psoriasis other than biological products, *n* (%)	Yes	751 (76.95)
Phototherapy (for PsA and psoriasis vulgaris)		
IGA score, *n* (%)	0 (clear)	35 (3.59)
	1 (almost clear)	48 (4.92)
	2 (mild)	162 (16.60)
	3 (moderate)	281 (28.79)
	4 (severe)	117 (11.99)
	Unknown/not recorded	333 (34.12)
PASI score	≤20	532 (54.51)
	>20	143 (14.65)
	Unknown/not recorded	301 (30.84)

Abbreviations: BMI, body mass index; IGA, Investigator's Global Assessment; *n*, number of applicable patients; *N*, total number of patients; PASI, Psoriasis Area and Severity Index; PsA, psoriatic arthritis; SD, standard deviation.

The majority of the patients (90.98%, *n* = 888/976) received other medications for psoriasis vulgaris prior to secukinumab treatment. Biologics were received by 42.52% of patients (*n* = 415/976), while 76.95% of patients (*n* = 751/976) received medications other than biologics. The most common reasons for switching from a prior biologic to secukinumab were lack of effectiveness; >70% of patients previously on adalimumab or ustekinumab switched to secukinumab due to this reason (Supporting Information Table S1). The most commonly used psoriasis medication other than biologics was topical corticosteroids (48.36%, *n* = 472/976), followed by cyclosporine (5.12%, *n* = 50/976). Patients who had received phototherapy for psoriasis vulgaris or PsA before the start of secukinumab treatment accounted for 20.80% (*n* = 203/976), and narrowband ultraviolet B (UVB) was the most common type of treatment (16.09%, *n* = 157/976). Excluding unknown or those not recorded, the most common IGA score at the start of secukinumab treatment was 3 (moderate) in 28.79% (*n* = 281/976), 2 (mild) in 16.60% (*n* = 162/976), and 4 (severe) in 11.99% (*n* = 117/976) of patients. The PASI score at the start of secukinumab treatment was ≤20 in 54.51% (*n* = 532/976) and >20 in 14.65% (*n* = 143/976) (Table 1).

### 3.2 Secukinumab administration and surveillance discontinuation

The mean ± standard deviation (SD) observation duration was 346.8 ± 61.81 days, and 90.88% (*n* = 887/976) of the population had an observation duration longer than 48 weeks. The mean ± SD duration of secukinumab administration was 288.1 ± 106.51 days, and the median (range) was 344.0 (1–365) days. A duration of secukinumab administration was >4 weeks for 95.70% (*n* = 934/976), >16 weeks for 87.09% (*n* = 850/976), and >48 weeks for 61.78% (*n* = 603/976). The initial secukinumab dose per administration was 300 mg in 97.44% (*n* = 951/976), and the most used secukinumab dose per administration was 300 mg (96.21%, *n* = 939/976). The median (range) total number of administrations was 16.0 (1–24). During the observation period, 72.03% (*n* = 703/976) of patients performed self‐administration at least once (Table 2). The surveillance was discontinued in 9.53% (*n* = 93/976) of patients, with the most common reason for discontinuation being “failure to visit in the middle of course (including hospital transfer)” in 6.66% (*n* = 65/976) (Table 3).

**TABLE 2:** Administration during the study period (safety analysis set).

Variable	Category/summary statistics	*N* = 976
Observation duration,^a^ *n* (%)	>4 weeks	973 (99.69)
	>16 weeks	954 (97.75)
	>24 weeks	939 (96.21)
	>32 weeks	904 (92.62)
	>40 weeks	896 (91.80)
	>48 weeks	887 (90.88)
	>52 weeks	884 (90.57)
Observation duration (day)	Mean ± SD	346.8 ± 61.81
	Median (range)	365.0 (21–365)
	Total observation duration (patient‐year)	926.8
Secukinumab treatment duration,^b^ *n* (%)	>4 weeks	934 (95.70)
	>16 weeks	850 (87.09)
	>24 weeks	799 (81.86)
	>32 weeks	732 (75.00)
	>40 weeks	689 (70.59)
	>48 weeks	603 (61.78)
	>52 weeks	196 (20.08)
	Unknown/not recorded	17 (1.74)
Secukinumab treatment duration (days), *n* = 959	Mean ± SD	288.1 ± 106.51
	Median (range)	344.0 (1–365)
	Total exposure duration (person‐years)	756.4
Starting dose of secukinumab (per administration), *n* (%)	150 mg	25 (2.56)
	300 mg	951 (97.44)
Most frequent dose of secukinumab (per administration), *n* (%)	150 mg	34 (3.48)
	300 mg	939 (96.21)
	Unknown/not recorded	3 (0.31)
Total number of secukinumab administration, *n* = 973	Mean ± SD	13.6 ± 3.87
	Median (range)	16.0 (1–24)
Secukinumab dose increase/reduction, *n* (%)	No dose increase/reduction	929 (95.18)
	Dose increase only	8 (0.82)
	Dose reduction only	26 (2.66)
	Dose increase and reduction	10 (1.02)
	Unknown/not recorded	3 (0.31)
Self‐administration,^c^ *n* (%)	No	272 (27.87)
	Yes	703 (72.03)
	Unknown/not recorded	1 (0.10)

Abbreviations: *n*, number of applicable patients; *N*, total number of patients; SD, standard deviation.

^a^ Observation duration = last observation day — first treatment day + 1.

^b^ Secukinumab administration duration = last administration day — first administration day + 1.

^c^ Patients who performed self‐administration at least once during the secukinumab administration period were classified to “Yes.”

**TABLE 3:** Number of discontinued patients and reasons for discontinuation (safety analysis set).

Breakdown	Number of patients, *n* (%) *N* = 976
52‐week observation completed	877 (89.86)
52‐week observation not completed^a^	99 (10.14)
Not completed^b^	6 (0.61)
Patients who discontinued	93 (9.53)
Reasons for discontinuation	
Failure to return before completion (including hospital change)	65 (6.66)
Withdrawal of consent by the patient	18 (1.84)
Death	8 (0.82)
Others	1 (0.10)
Unknown	1 (0.10)

*Note*: Patient with multiple reasons for discontinuation are counted in the respective reasons.

Abbreviations: *n*, number of applicable patients; *N*, total number of patients.

^a^ Patients who did not complete the 52‐week observation (including patients who discontinued and patients who did not complete).

^b^ Patients whose second surveillance form was not collected or patients whose second surveillance form data were excluded from analysis.

### 3.3 Safety outcomes

The safety analysis of the population (*n* = 976) was evaluated. Incidences of AEs, SAEs, and AEs suspected to be related to secukinumab are summarized in Table 4. AEs were reported in 353 patients (36.17%). The AEs (≥2% patients) included nasopharyngitis (3.07%, *n* = 30/976), psoriatic arthropathy (2.97%, *n* = 29/976), psoriasis (2.66%, *n* = 26/976), and oral candidiasis (2.05%, *n* = 20/976).

**TABLE 4:** Incidence of AEs, SAEs, and AEs suspected to be related to secukinumab (safety analysis set).

Safety analysis population	*n* (%) *N* = 976
Number of patients with at least one AE	353 (36.17)
Number of patients with at least one SAE	57 (5.84)
Number of patients with at least one AR	184 (18.85)
AEs that led to secukinumab treatment discontinuation	79 (8.09)
AEs that led to treatment discontinuation with a history of biologics use (*n* = 413)	43 (10.41)
AEs that led to treatment discontinuation without a history of biologics use (*n* = 559)	36 (6.44)
Death	11 (1.13)

Abbreviations: AE, adverse event; AR, adverse reaction; *n*, number of patients with at least one event; *N*, total number of patients; SAE, serious adverse event.

The safety evaluations included 413 patients with a history of biologics use and 559 patients without a history of biologics use, and patients with a history of biologics use had higher incidences of AEs (40.44%, *n* = 167/413) than those without a history of biologics use (33.27%, *n* = 186/559). No significant difference was seen in the incidence and type of AEs between the patients with and without a history of biologics use. AEs suspected to be related to secukinumab were reported in 184 patients (18.85%). The AEs suspected to be related to secukinumab (≥1% patients) included oral candidiasis (1.95%, *n* = 19/976), psoriatic arthropathy (verbatim: worsening of underlying disease, etc., 1.33%, *n* = 13/976), pruritus (1.23%, *n* = 12/976), and psoriasis (verbatim: worsening of underlying disease, etc., 1.13%, *n* = 11/976). Patients with the current medical conditions of hypersensitivity reactions (31.82% vs. 18.31%, OR 2.08, 95% CI 1.00, 4.15) and those with cardiovascular/cerebrovascular events (27.72% vs. 16.93%, OR 1.88, 95% CI 1.27, 2.77) had higher incidence of AEs suspected to be related to secukinumab. After adjusting patient characteristics suspected to be clinically related, cardiovascular/cerebrovascular events had an adjusted OR of 1.70 with 95% CI (1.11, 2.60) that did not contain 1 (Supporting Information Figure S1).

AEs that led to secukinumab treatment discontinuation were reported in 79 patients (8.09%) and common AEs were psoriasis (verbatim: worsening of underlying disease, etc., 1.33%, *n* = 13/976), psoriatic arthropathy (verbatim: worsening of underlying disease, etc., 1.02%, *n* = 10/976), and ineffectiveness (0.51%, *n* = 5/976) (Table 4).

SAEs were reported in 57 patients (5.84%). SAEs occurring in ≥2 patients included cellulitis (0.41%, *n* = 4/976), herpes zoster, pneumonia, myocardial infarction, and interstitial lung disease (0.31%, *n* = 3/976 each), and septic shock, hyperkalemia, and cerebral infarction (0.20%, *n* = 2/976 each). SAEs were reported in 6.30% of patients (*n* = 26/413) with a history of biologics use and in 5.55% of patients (*n* = 31/559) without a history of biologics use. Most of these SAEs occurred only once. Similar SAE profiles were observed between patients with a history of biologics use (6.30%) and those without (5.55%).

Of 11 deaths (1.13%) that were reported during the safety analysis period, the causal relationship of the deaths was ruled out for nine patients. The fatal events suspected by the investigators to be related to secukinumab were death and toxic skin eruption in one patient each. The patient (preferred term [PT]: death) was a 69‐year‐old male with neither a medical history nor a history of previous biologics use. Renal disease was reported as a current condition. Platelet count decreased (severe) about 2 months before death (date of occurrence unknown), and the outcome was unknown. Secukinumab administration was discontinued after the onset of decreased platelet. This patient died 314 days after the start of secukinumab treatment (34 days from the most recent administration day). The patient (PT: toxic skin eruption) was a 76‐year‐old male without a medical history and with a history of previous biologics use who had current medical conditions. Toxic skin eruption (moderate severity) occurred on the fifth day from the first secukinumab administration. Secukinumab administration was discontinued after the occurrence. The outcome was fatal (368 days from the occurrence). Pneumonia (serious event) occurred 330 days after the start of treatment, and the patient died 43 days after the onset. Pneumonia was considered not related to secukinumab administration. The cause other than secukinumab was complications.

The incidence of priority investigation items (AEs and AEs suspected to be related to secukinumab) during the observation period are shown in Table 5. The priority investigation items (≥0.50%) reported as AEs suspected to be related to secukinumab included oral candidiasis (1.95%, *n* = 19/976), tinea pedis (0.92%, *n* = 9/976), and rash (0.51%, *n* = 5/976). Each priority investigation item is described in the sections below. No tuberculosis was reported.

**TABLE 5:** Incidence of priority investigation items (≥0.3% of patients) (safety analysis set).

Priority investigation items PT	Safety analysis population *N* = 976			
	AEs *n* (%)		ARs *n* (%)	
Total	134	(13.73)	74	(7.58)
Serious infections	17	(1.74)	9	(0.92)
Cellulitis	4	(0.41)	3	(0.31)
Herpes zoster	3	(0.31)	2	(0.20)
Pneumonia	3	(0.31)	0	(0.00)
Fungal infections	54	(5.53)	41	(4.20)
Oral candidiasis	20	(2.05)	19	(1.95)
Tinea pedis	19	(1.95)	9	(0.92)
Body tinea	5	(0.51)	4	(0.41)
Tuberculosis	0	(0.00)	0	(0.00)
Neutropenia	6	(0.61)	3	(0.31)
White blood cell count decreased	4	(0.41)	1	(0.10)
Neutrophil count decreased	3	(0.31)	3	(0.31)
Hypersensitivity reactions	43	(4.41)	14	(1.43)
Rash	9	(0.92)	5	(0.51)
Urticaria	8	(0.82)	2	(0.20)
Eczema	7	(0.72)	3	(0.31)
Dermatitis contact	5	(0.51)	0	(0.00)
Rhinitis allergic	4	(0.41)	0	(0.00)
Toxic skin eruption	3	(0.31)	2	(0.20)
Malignant tumors	7	(0.72)	3	(0.31)
Inflammatory bowel disease	2	(0.20)	2	(0.20)
Cardiovascular/cerebrovascular events	21	(2.15)	5	(0.51)
Hypertension	5	(0.51)	0	(0.00)
Myocardial infarction	3	(0.31)	0	(0.00)

*Note*: Multiple episodes of an event (PT) in the same patient are counted only once. Priority investigation items (order of appearance in the surveillance form); PT codes are shown in descending order of incidences in the AE column. MedDRA/J version 24.1.

Abbreviations: AE, adverse event; AR, adverse reaction; *n*, number of applicable patients; *N*, total number of patients; PT, preferred term.

### 3.4 Serious infections

Serious infections were reported in 1.74% of patients (*n* = 17/976) as AEs and in 0.92% of patients (*n* = 9/976) as AEs suspected to be related to secukinumab. The median (range) number of days from the start of secukinumab treatment to the occurrence of AEs suspected to be related to secukinumab (first occurrence) was 81.0 (1–308) days. Events reported as severe were herpes zoster and septic shock in one patient each; all other events were moderate in severity. The outcomes in the nine patients with AEs suspected to be related to secukinumab were all resolved or resolving except for sequelae (herpes zoster). The median (range) number of days to resolved status or resolving was 21.0 (1–113) days. Among the serious infections that occurred during the follow‐up period, the AEs suspected to be related to secukinumab were psoas abscess (mild), pneumonia (severe), and appendicitis (mild) in one patient each. Serious infections that occurred during the follow‐up period were similar to the events that occurred within the observation period, not showing a tendency toward increased occurrence of serious infections with long‐term secukinumab treatment.

### 3.5 Neutropenia

Neutropenia was reported in 0.61% (*n* = 6/976) as an AE and in 0.31% (*n* = 3/976) as an AE suspected to be related to secukinumab. The AEs of neutropenia included decreased white blood cell count (0.41%, *n* = 4/976) and decreased neutrophil count (0.31%, *n* = 3/976). The AEs suspected to be related to secukinumab of neutropenia included decreased neutrophil count (0.31%, *n* = 3/976) and decreased white blood cell count (0.10%, *n* = 1/976). The median (range) number of days from the start of secukinumab treatment to the occurrence of AEs suspected to be related to secukinumab (first occurrence) was 29.0 (29–113) days. The severity of the events was mild, except for one patient with unknown severity for decreased white blood cell and neutrophil count. The median (range) number of days to AE resolved or resolving was 511.5 (351–672) days.

### 3.6 Fungal infections

Fungal infections were reported in 5.53% (*n* = 54/976) as an AE and in 4.20% (*n* = 41/976) as an AE suspected to be related to secukinumab. The most common AE and the AE suspected to be related to secukinumab of fungal infections were oral candidiasis in 20 (2.05%) and 19 (1.95%) patients, respectively. The median (range) number of days from the start of secukinumab treatment to the occurrence of AEs suspected to be related to secukinumab (first occurrence) was 71.0 (10–359) days. All events were mild or moderate in severity. The outcomes in 41 patients were all resolved or resolving, except for one patient each with unknown outcomes (tinea pedis, tinea cruris). The median (range) number of days to resolved or resolving was 85.0 (7–843) days.

### 3.7 Hypersensitivity reactions

Hypersensitivity reactions were reported in 4.41% (*n* = 43/976) as an AE and in 1.43% (*n* = 14/976) as an AE suspected to be related to secukinumab. The most common AE and the AE suspected to be related to secukinumab of hypersensitivity reactions were rash in nine (0.92%) and five patients (0.51%), respectively. The median (range) number of days from the start of secukinumab treatment to the occurrence of AEs suspected to be related to secukinumab (first occurrence) was 165.0 (5–337) days. The severity of events was either mild or moderate, except for one patient who had severe eczema and one patient with unknown severity of dermatitis psoriasiform. The outcomes in the 14 patients were all resolved or resolving, except for one patient each with the outcomes not resolved and unknown (two cases of eczema: same patient), not resolved (rash), and death (toxic skin eruption). The median (range) number of days to resolved or resolving was 35.5 (16–143) days (number of days could not be calculated for one of the “resolved” patients because of the unknown date of occurrence).

### 3.8 Malignant tumors

Malignant tumors were reported in 0.72% (*n* = 7/976) as an AE and in 0.31% (*n* = 3/976) as an AE suspected to be related to secukinumab. The median (range) number of days from the start of secukinumab treatment to the occurrence of AEs suspected to be related to secukinumab (first occurrence) were 260.0 (133–305) days. The severity of ovarian cancer and malignant neoplasm of unknown primary site was severe. The severity of pleomorphic adenoma was unknown. The outcomes after actions (interruption of secukinumab, secukinumab treatment discontinuation, hospitalization/prolongation of current hospitalization) were resolving. The median (range) number of days to resolving was 268.0 (114–524) days. Bladder cancer, pancreatic cancer, and malignant neoplasm of the lung were reported in one patient each during the follow‐up period.

### 3.9 Inflammatory bowel disease

Incidence of IBD for both AEs and AEs suspected to be related to secukinumab was reported in two patients (0.20%) each. In one patient with IBD (PT), there was a complication, and the complication was not related to the study drug. In the other patient there was no complication of IBD. The observed AEs suspected to be related to secukinumab were colitis ulcerative and IBD [PT] in one patient (0.10%) each; both were assessed to be serious and moderate in severity with the outcome resolved. The number of days for the occurrence of colitis ulcerative (worsening of the primary disease) was 228 days, and the number of days taken to resolve was 131 days. The date of occurrence of the IBD (PT) was unknown, and the number of days taken to resolve could not be calculated.

### 3.10 Cardiovascular/cerebrovascular events

Cardiovascular/cerebrovascular events were reported in 2.15% (*n* = 21/976) as AEs and in 0.51% (*n* = 5/976) as AEs suspected to be related to secukinumab. The severity was severe in one patient (death), moderate in one patient (cerebral infarction), and mild in all other events. The median (range) number of days from the start of secukinumab treatment to the first occurrence of AEs suspected to be related to secukinumab was 171.5 (86–314) days. The AEs suspected to be related to secukinumab resolved in two of the five patients, and the median (range) number of days to resolved was 47.5 (1–94) days.

### 3.11 Effectiveness outcomes

**FIGURE 2:**
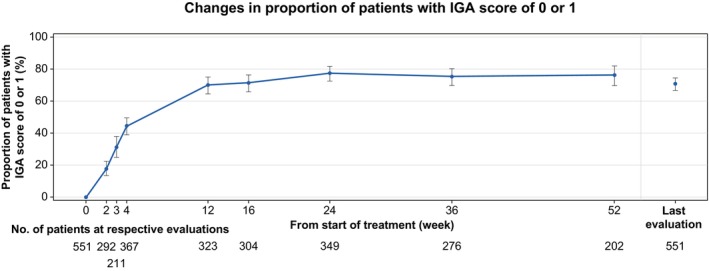
Changes over time in the proportion of patients with an IGA score of 0 or 1 (effectiveness analysis set). The proportion of patients with an IGA score of 0 or 1 and the 95% CIs are shown. The Clopper–Pearson method was used for the calculation of 95% CI. Last evaluation: Last time point with a result during 52 weeks of observational period. Population: Patients with an IGA score of >2 at the start of treatment and with the last evaluation present. CI, confidence interval; IGA, Investigator's Global Assessment.

The effectiveness analysis population (*n* = 729) was evaluated. IGA score assessment was performed for patients from the effectiveness analysis population whose IGA score was ≥2 at the start of secukinumab treatment. The proportion of patients with an IGA score improvement to 0 or 1 after the start of secukinumab treatment increased over time, reaching 44.41% (*n* = 163/367) at week 4, 71.38% (*n* = 217/304) at week 16, and 77.36% (*n* = 270/349) at week 24 (Figure 2). Thereafter, the proportion of patients with IGA 0/1 response remained stable, with 76.24% (*n* = 154/202) at week 52. For 202 patients with a baseline IGA score of ≥2 at the start of secukinumab treatment, the response rates were analyzed by patient characteristics. The response rate in patients with renal impairment was 52.94% versus 78.65% in patients without renal impairment. Although the effectiveness tended to be lower in patients with renal impairment, the OR of the response rate was such that the upper limit of 95% CI was slightly lower than 1 (OR 0.31, 95% CI 0.10, 0.98), and the multivariate logistic regression analysis suggested the influence of other factors (OR 0.39, 95% CI 0.08, 2.36), therefore, this was not considered to be investigated as a factor likely to affect efficacy (Supporting Information Figure S2).

**FIGURE 3:**
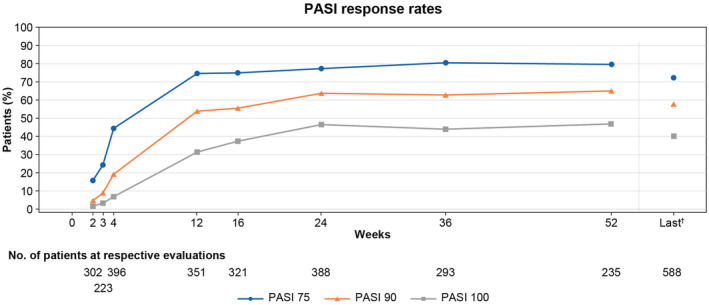
PASI 75/90/100 response rates (effectiveness analysis set). ^†^Last time point with a result during 52 weeks of observational period. Included are patients with results at the start of secukinumab treatment and the last measurement. PASI, Psoriasis Area and Severity Index.

The PASI 75 response rate increased over time from week 2 to week 12 of treatment and became stable thereafter up to week 52. The PASI 90 and PASI 100 response rates increased over time from week 2 to week 24 of treatment and became stable thereafter up to week 52. The PASI 75 response rates at weeks 16 and 52 were 73.83% (*n* = 237/321) and 78.30% (*n* = 184/235), the PASI 90 response rates were 55.14% (*n* = 177/321) and 64.26% (*n* = 151/235), and the PASI 100 response rates were 37.69% (*n* = 121/321) and 46.81% (*n* = 110/235), respectively (Figure 3).

**FIGURE 4:**
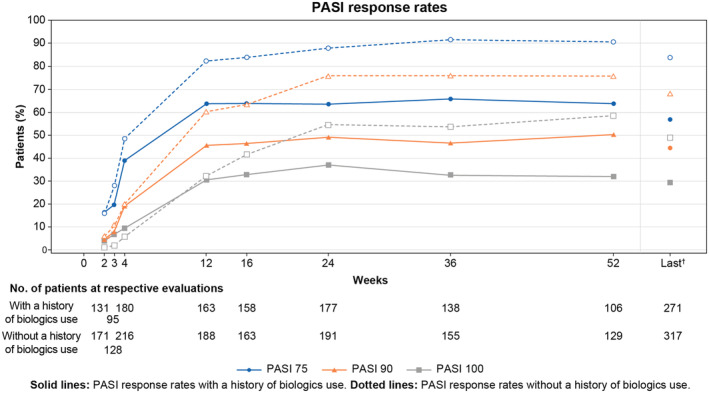
PASI 75/90/100 response rates by presence/absence of a history of biologics use prior to the start of secukinumab treatment (effectiveness analysis set). ^†^Last time point with a result during 52 weeks of observation. PASI, Psoriasis Area and Severity Index.

The PASI response rates at week 12 from the start of secukinumab treatment, at week 16, and at week 52 in the patients with a history of biologics use showed increasing trends with time until week 16 and were maintained up to week 52: the PASI 75 response rates were 63.19%, 63.29%, and 63.21%; the PASI 90 response rates were 45.40%, 46.20%, and 50.00%; and the PASI 100 response rates were 30.67%, 32.91%, and 32.08% at weeks 12, 16, and 52, respectively. The PASI response rates in the patients with a history of biologics use tended to be lower than those in patients without a history of biologics use. There were no significant differences in PASI response rates by number of days to a switch to secukinumab from previous biologics (≤12 weeks vs. >12 weeks) (Figure 4).

The mean ± SD DLQI total score was 7.4 ± 6.13 at the start of secukinumab treatment, 3.7 ± 4.57 at week 4, 2.1 ± 3.56 at week 16, and 1.8 ± 2.54 at week 52. The proportions of patients with a DLQI total score of 0 or 1 at the respective evaluation time points were 14.79% (*n* = 63/426) at the start of secukinumab treatment, 40.25% (*n* = 130/323) at week 4, 66.67% (*n* = 162/243) at week 16, 68.64% (*n* = 116/169) at week 36, and 63.56% (*n* = 75/118) at week 52 (Supporting Information Figure S3).

The mean scores of HAQ‐DI, DAS28‐CRP, VAS (physician and patient), dactylitis count in fingers and toes, tender/swollen DIP joint count, and BASDAI decreased over time from the start of secukinumab treatment and remained stable from week 12 to week 52. Evaluation of the presence or absence of enthesis tenderness (Leeds) in patients showed that the proportion of patients with at least one symptom decreased from week 4 to week 52. In Global Impression of Change of joint symptoms, the response rates were calculated, defining complete response and partial response as “responded.” At least 80% of patients responded at week 4 from the start of secukinumab treatment, and the response rate remained stable at this level up to week 52 (Table 6).

**TABLE 6:** Disease‐related assessment in patients with PsA (patients with PsA in the effectiveness analysis set).

Assessment	Baseline	Week 4	Week 12	Week 16	Week 24	Week 52	Last evaluation
HAQ‐DI (mean changes ± SD)	NA	−0.16 ± 0.408 (*n* = 85)	−0.20 ± 0.527 (*n* = 70)	−0.17 ± 0.511 (*n* = 64)	−0.27 ± 0.601 (*n* = 66)	−0.31 ± 0.595 (*n* = 38)	−0.26 ± 0.589 (*n* = 107)
DAS28‐CRP (mean changes ± SD)	NA	−0.87 ± 0.875 (*n* = 17)	−1.08 ± 1.370 (*n* = 64)	−1.33 ± 1.297 (*n* = 48)	−1.21 ± 1.437 (*n* = 64)	−1.20 ± 1.526 (*n* = 37)	−1.13 ± 1.398 (*n* = 111)
VAS (physician) (mean changes ± SD)	NA	−28.2 ± 23.82 (*n* = 100)	−29.6 ± 31.12 (*n* = 89)	−37.5 ± 28.44 (*n* = 79)	−34.8 ± 30.73 (*n* = 84)	−38.5 ± 31.24 (*n* = 47)	−31.4 ± 29.75 (*n* = 139)
VAS (patient) (mean changes ± SD)	NA	−17.7 ± 27.10 (*n* = 105)	−20.5 ± 33.03 (*n* = 94)	−24.6 ± 32.98 (*n* = 87)	−24.7 ± 33.85 (*n* = 89)	−21.4 ± 37.02 (*n* = 52)	−18.4 ± 35.53 (*n* = 142)
Dactylitis count^a^ (mean changes ± SD)	NA	−0.7 ± 1.97 (*n* = 107)	−1.3 ± 4.06 (*n* = 94)	−2.3 ± 4.98 (*n* = 85)	−2.1 ± 4.68 (*n* = 93)	−1.8 ± 4.69 (*n* = 57)	−1.5 ± 3.98 (*n* = 147)
Tender DIP joints (mean changes ± SD)	NA	−1.1 ± 2.88 (*n* = 103)	−1.3 ± 3.75 (*n* = 93)	−2.2 ± 4.05 (*n* = 86)	−1.4 ± 3.59 (*n* = 94)	−1.7 ± 4.45 (*n* = 55)	−1.4 ± 3.83 (*n* = 147)
Swollen DIP joints (mean changes ± SD)	NA	−0.6 ± 1.79 (*n* = 102)	−0.7 ± 2.60 (*n* = 95)	−1.1 ± 3.71 (*n* = 84)	−0.8 ± 2.72 (*n* = 95)	−1.4 ± 3.29 (*n* = 55)	−1.1 ± 2.96 (*n* = 145)
BASDAI (mean changes ± SD)	NA	−1.28 ± 1.842 (*n* = 94)	−1.62 ± 1.956 (*n* = 82)	−1.77 ± 1.967 (*n* = 78)	−1.78 ± 2.224 (*n* = 79)	−2.03 ± 2.344 (*n* = 43)	−1.63 ± 2.165 (*n* = 129)
Tenderness in entheses (Leeds), % (*n*/*M*)	32.07 (59/184)	25.56 (34/133)	17.56 (23/131)	22.52 (25/111)	13.53 (18/133)	10.23 (9/88)	12.85 (23/179)
Global Impression of Change of joint symptoms (Response rate)^b^, % (*n*/*M*)	NA	80.33 (98/122)	79.13 (91/115)	85.71 (84/98)	84.91 (90/106)	77.33 (58/75)	76.27 (135/177)

Abbreviations: BASDAI, Bath Ankylosing Spondylitis Disease Activity Index; CRP, C‐reactive protein; DAS 28, Disease Activity Score 28; DIP, distal interphalangeal; HAQ‐DI, Health Assessment Questionnaire©—Disability Index; *M*, total number of patients assessed; *n*, number of patients; NA, not applicable; PsA, psoriatic arthritis; SD, standard deviation; VAS, visual analog scale.

^a^ Fingers and toes.

^b^ Patients were defined as having “responded” if they had a complete response and a partial response.

## 4 DISCUSSION

This surveillance evaluated the safety and effectiveness of secukinumab use until week 52 and followed up to 156 weeks from the start of secukinumab treatment. Long‐term safety and effectiveness of secukinumab in psoriasis, PsA, and ankylosing spondylitis were reported in several previous studies.^11^, ^12^, ^13^, ^14^ Common AEs of infections and infestations, respiratory, thoracic and mediastinal disorders, and skin and subcutaneous tissue disorders are consistent with the results in a recent post‐marketing data mining study of the US FDA AE reporting system.^15^ The observed incidences of AEs (36.17%) and AEs suspected to be related to secukinumab (18.85%) were lower than those in the previous clinical studies.^6^, ^16^, ^17^ The incidence of SAEs (5.84%) was comparable with that in other studies, and a similar trend was observed between this surveillance and previous clinical studies.^6^, ^16^

The AEs and AEs suspected to be related to secukinumab of the priority investigation items were consistent with previous reports, with no new safety signals reported.^6^, ^17^ In this surveillance, the outcomes in the nine patients with serious infections (AEs suspected to be related to secukinumab) were all resolved or resolving, except for sequelae due to herpes zoster. Increased risk of serious infections was reported in pooled analyses as potentially related to prior biologics usage and the immune dysregulations of psoriasis.^11^, ^12^ The incidence of AEs from a pooled analysis was comparable to the current results, and secukinumab treatment was not related to the occurrence of IBD.^18^ The outcomes in three patients with malignant tumors (AEs suspected to be related to secukinumab) were all resolving, and no safety concern was identified. The data on the occurrence of AEs, SAEs, AR, AEs leading to treatment discontinuation, and AEs suspected to be related to secukinumab of the priority investigation items showed similar profiles by history of biologics.

Psoriasis and PsA are closely associated with cardiovascular diseases.^19^ The analysis showed that AEs suspected to be related to secukinumab occurred more frequently in patients with cardiovascular comorbidities. By system organ class, the most common AEs suspected to be related to secukinumab in patients with cardiovascular/cerebrovascular events were infections and infestations. Seven serious infections in patients with cardiovascular/cerebrovascular events were all associated with possible non‐secukinumab factors (underlying disease, current medical condition [diabetes mellitus or chronic cystitis], and concomitant drug [prednisolone]), due to which it is not possible to identify the presence or absence of cardiovascular/cerebrovascular events as a risk factor of serious infections. Although the incidences of cellulitis, nasopharyngitis, skin candida, malaise, and pyrexia were higher in the patients with cardiovascular/cerebrovascular events, the outcomes were all resolved or resolving and no new concern requiring attention calling was detected.

This surveillance supports the efficacy data from a phase 3 Japanese data and is of a larger scale than other phase 4 Japanese studies or real‐world studies.^6^, ^16^, ^17^, ^20^ In the effectiveness analysis, a favorable outcome with PASI 75/90/100 responses after secukinumab treatment is comparable with previous clinical trials.^16^, ^21^ The response rate reported in this surveillance is also comparable to a real‐world evidence study by Fujita et al. where >80% of patients responded by week 4 and remained stable up to week 24.^6^ In addition, lower PASI response rates were observed in patients with a history of biologics use, suggesting that secukinumab was more effective in patients with no prior treatment with biologics.^17^ In a retrospective study in Japan, no significant differences were observed in PASI 75 and 90 responses after secukinumab treatment between patients with a history of biologics and those with no prior treatment with biologics at weeks 12, 24, and 52, but numerically higher responses were observed in patients who switched from other biologics.^20^ The proportion of patients with an IGA score improvement to 0 or 1 increased over time from the start of secukinumab treatment to week 24 and was stable thereafter. The proportion of patients with an IGA score improvement in this surveillance (69.97% [226 of 323 patients] at week 12 and 76.24% [154 of 202 patients] at week 52) was similar with previous clinical study.^17^ In line with previous reports, the quality of life for patients also improved with secukinumab treatment, which was evidenced by the sustained improvement in DLQI scores.^10^, ^13^, ^16^

The effectiveness of secukinumab was also proven from the improvements in HAQ‐DI scores, DAS28‐CRP scores, VAS (physician), VAS (patient), dactylitis count in fingers and toes, tender DIP joint count, swollen DIP joint count, tenderness in entheses, presence/absence of dactylitis in fingers and toes, and BASDAI scores over time from the start of secukinumab treatment, and the improvements tended to be maintained up to week 52. A mean decrease of 0.31 in HAQ‐DI score from the start of secukinumab treatment to week 52 showed a clinically significant change, and the decrease was consistent with the results from a previous study.^22^ These data show that the long‐term effectiveness of secukinumab in clinical use in patients with psoriasis vulgaris or PsA not adequately responding to conventional therapies was similar to previous studies.^21^, ^23^, ^24^

This surveillance was an observational study without a control group and did not collect information on patients not exposed to secukinumab, which is the limitation for the postulation of the causality between secukinumab exposure and the observed results. The results from this surveillance were compared using previous studies for reference, but there are differences between the studies in terms of the patient population and study design. In addition, other limitations included incomplete data and confounding factors that could not be identified due to the “unknown” answers.

In conclusion, the surveillance results confirm the long‐term effectiveness of secukinumab treatment in patients with psoriasis vulgaris and PsA. No new safety concern of long‐term secukinumab treatment was reported.

## CONFLICT OF INTEREST STATEMENT

A.F. and N.S. are employees of Novartis Pharma K.K. There were no financial or personal relationships between authors and others that could bias the work set out in the manuscript, as declared in the Conflict of Interest Statement.

## ETHICS STATEMENT

The study followed the ethical policies of this Journal and the institution's ethics committees.

## Supporting information


Data S1.
