# Supplementary material for: Safety and effectiveness of secukinumab subcutaneous injection in Japanese patients with psoriasis vulgaris and psoriatic arthritis: A post‐marketing surveillance
Source: J Dermatol. 2024 Nov 29;52(1):11–23. doi: 10.1111/1346-8138.17499 (PMC11700941; doi:10.1111/1346-8138.17499)
Supplement: Supplementary file 1 — Data S1. [file JDE-52-11-s001.docx]

###### Table S1. Reasons for a switch by type of biologics used prior to the start of secukinumab treatment (safety analysis population)

| **Drug names** | **Reasons for a switch** | | | | |
| --- | --- | --- | --- | --- | --- |
|  | **Lack of efficacy**  **n (%)** | **Adverse events**  **n (%)** | **Patient’s decision**  **n (%)** | **Financial reasons**  **n (%)** | **Others**  **n (%)** |
| Adalimumab (N = 198) | 141 (71.21) | 39 (19.70) | 10 (5.05) | 5 (2.53) | 6 (3.03) |
| Infliximab (N = 153) | 107 (69.93) | 34 (22.22) | 8 (5.23) | 1 (0.65) | 6 (3.92) |
| Ustekinumab (N = 168) | 152 (90.48) | 9 (5.36) | 3 (1.79) | 4 (2.38) | 1 (0.60) |
| Anti–IL-17 receptor antibody (N = 6) | 2 (33.33) | 1 (16.67) | 0 (0.00) | 1 (16.67) | 2 (33.33) |
| Others (N=20) | 11 (55.00) | 0 (0.00) | 4 (20.00) | 0 (0.00) | 4 (20.00) |

IL-17, interleukin-17; N, number of patients previously treated with; n, number of patients

Patients who had received more than one biologic are counted in the respective biologics.

Denominator for the proportions: number of patients with a history of use of each biologic.

###### Figure S1. Odds ratio of adverse reactions by patient characteristics (safety analysis set)


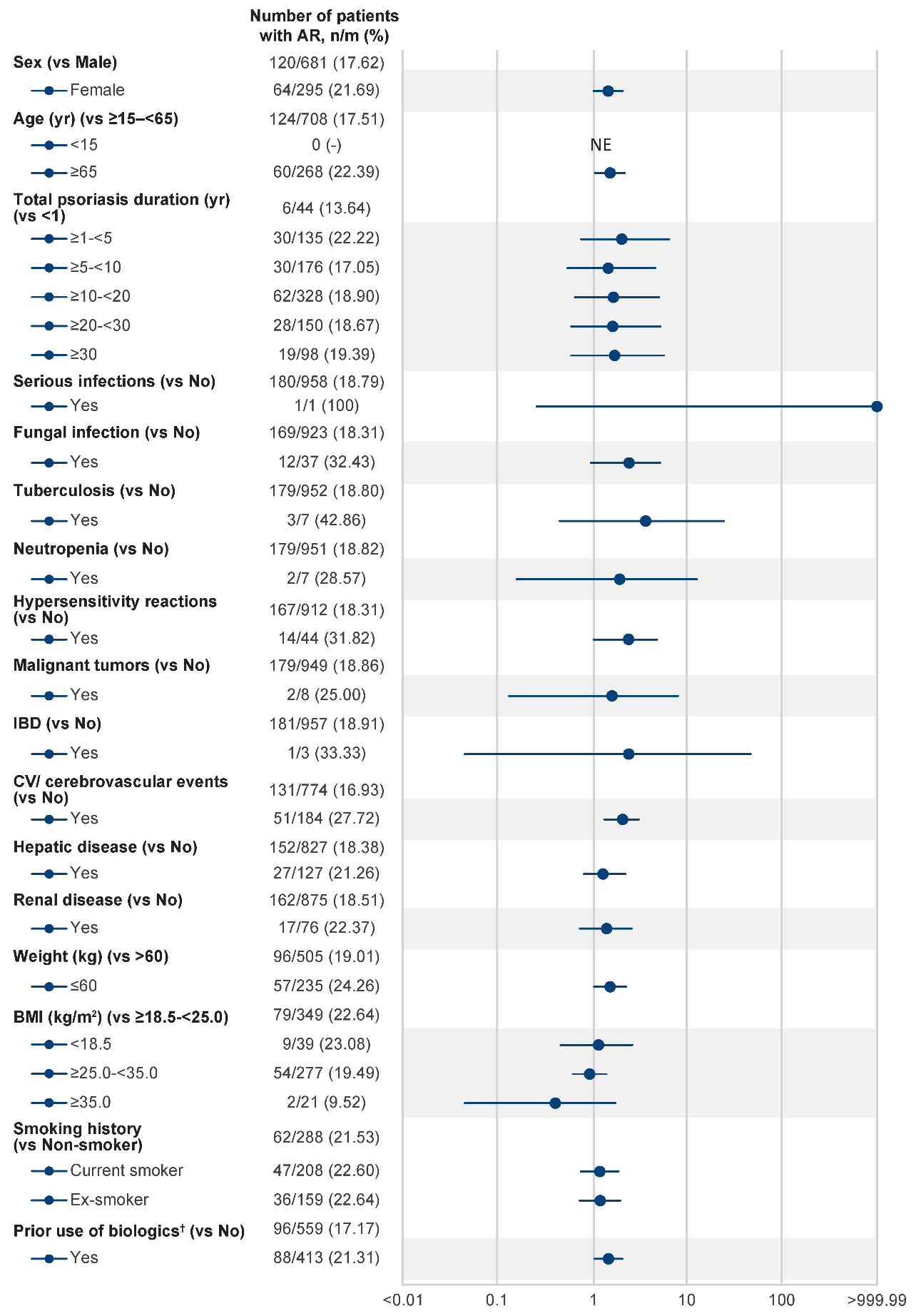


AR, adverse reaction; BMI, body mass index; CV, cardiovascular; IBD, inflammatory bowel disease; m, total number of patients in the respective category; n, number of applicable patients; NE, not estimable; PsA, psoriatic arthritis; yr, year.

^†^Use of biologics for psoriasis vulgaris or PsA prior to the start of secukinumab treatment.

###### Figure S2. Odds ratio of response rates by patient characteristics (IGA score improvement to 0 or 1 at week 52) (effectiveness analysis set)


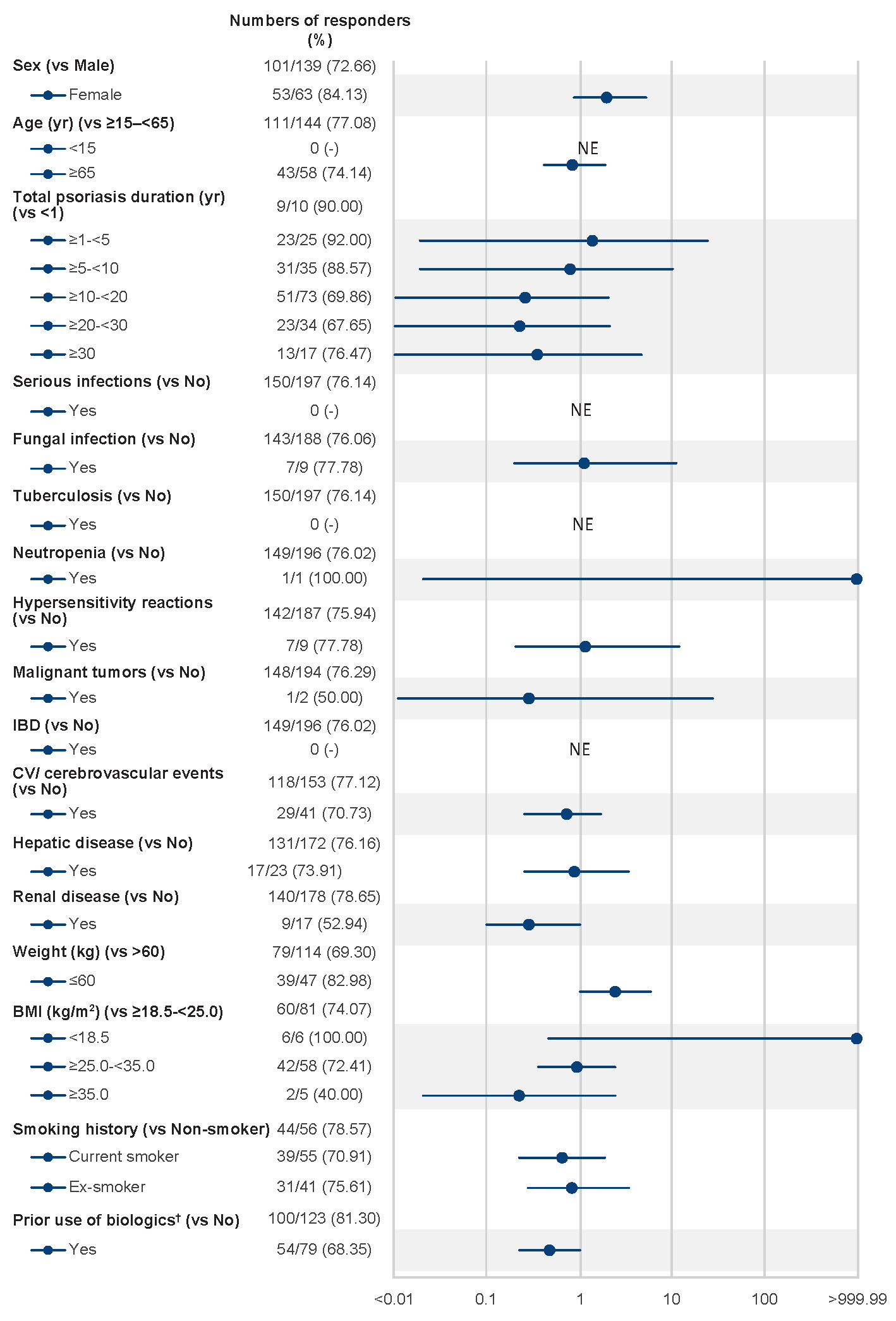


BMI, body mass index; CV, cardiovascular; IBD, inflammatory bowel disease; IGA, Investigator’s Global Assessment; m, total number of patients in the respective category; n, number of applicable patients; NE, not estimable; PsA, psoriatic arthritis; yr, year.

^†^Previous biologics for psoriasis vulgaris or PsA given prior to the start of secukinumab treatment.

###### Figure S3. Proportions of patients with a DLQI total score of 0 or 1 (effectiveness analysis set)


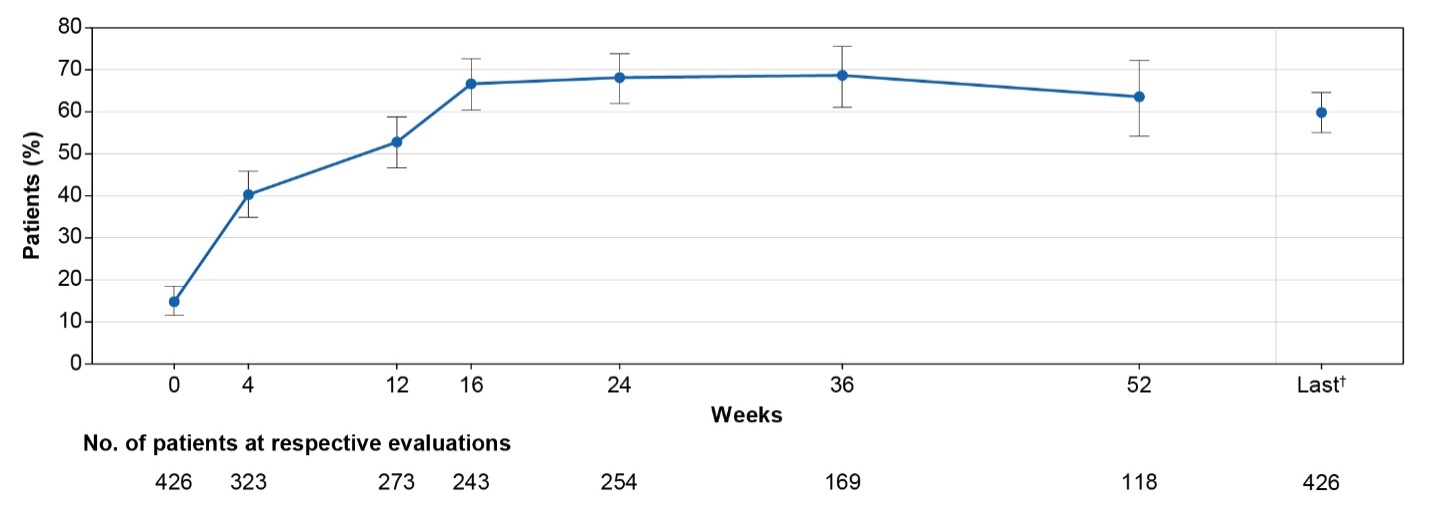


DLQI, Dermatology Life Quality Index.

^†^Last time point during 52 weeks of observational period.

Included are patients with results at the start of secukinumab and the last measurement. Proportion of patients with DLQI 0 or 1 and the 95% CIs were shown.
